# Efficient decellularization for tissue engineering of the tendon-bone interface with preservation of biomechanics

**DOI:** 10.1371/journal.pone.0171577

**Published:** 2017-02-07

**Authors:** Kai Xu, Lara A. Kuntz, Peter Foehr, Katharina Kuempel, Alexandra Wagner, Jutta Tuebel, Constantin V. Deimling, Rainer H. Burgkart

**Affiliations:** 1 Department of Orthopaedics and Sportsorthopaedics, Klinikum rechts der Isar, Technical University of Munich (TUM), Munich, Germany; 2 Department of Orthopedics, Tongji Hospital, Huazhong University of Science and Technology (HUST), Wuhan, China; Michigan Technological University, UNITED STATES

## Abstract

Interfaces between tendon/ligament and bone (“entheses”) are highly specialized tissues that allow for stress transfer between mechanically dissimilar materials. Entheses show very low regenerative capacity resulting in high incidences of failure after surgical repair. Tissue engineering is a promising approach to recover functionality of entheses. Here, we established a protocol to decellularize porcine entheses as scaffolds for enthesis tissue engineering. Chemical detergents as well as physical treatments were investigated with regard to their efficiency to decellularize 2 mm thick porcine Achilles tendon entheses. A two-phase approach was employed: study 1 investigated the effect of various concentrations of sodium dodecyl sulfate (SDS) and *t*-octylphenoxypolyethoxy-ethanol (Triton X-100) as decellularization agents. The most efficient combination of SDS and Triton was then carried forward into study 2, where different physical methods, including freeze-thaw cycles, ultrasound, perfusion, and hydrostatic washing were used to enhance the decellularization effect. Cell counts, DNA quantification, and histology showed that washing with 0.5% SDS + 1% Triton X-100 for 72 h at room temperature could remove ~ 98% cells from the interface. Further investigation of physical methods proved that washing under 200 mmHg hydrostatic pressure shortened the detergent exposing time from 72 h to 48 h. Biomechanical tensile testing showed that the biomechanical features of treated samples were preserved. Washing under 200 mmHg hydrostatic pressure with 0.5% SDS + 1% Triton X-100 for 48 h efficiently decellularized entheses with preservation of matrix structure and biomechanical features. This protocol can be used to efficiently decellularize entheses as scaffolds for tissue engineering.

## Introduction

“Entheses” are defined as the area where tendon or ligament insert into bone. These tissue interfaces allow smooth transmission of forces between two mechanically dissimilar tissues and minimize formation of stress peaks[1]. Entheses have been described as fibrous or fibrocartilaginous, according to the insertion of the tendon fibers into the bone tissue in indirect or direct way[2–4]. The term "enthesis" in this study is used for fibrocartilaginous entheses, which have been described to comprise four regions: tendon, fibrocartilage, mineralized fibrocartilage, and bone[4].

There is much interest in the repair of the entheses due to entheses’ poor capacity of regeneration[5,6]. Reconstruction of the enthesis following defects or damage is a great challenge for orthopedic surgeons. Previously, various treatments have been developed to fix the tendon or ligament to the bone involved using a suspending device through surgical operations or using biodegradable interference fit fixation. Despite appropriate medical management during such treatments, the graded transitional structure has yet to be successfully reconstructed[7,8]. Healing at the interface usually results in the formation of tissue with lower mechanical strength than before injury[9, 10]. Consequently, there is a high incidence of tendon pull-out and graft failure[11].

Several attempts have been made to improve repair of the enthesis by augmenting the strength of the ligament-bone graft fixation site in vivo including: growth factors, biomaterials, cells, and physical treatment options[12]. In case of manufacturing artificial tissues for implantation, polymeric scaffolds have been evaluated to be inserted as adjunct to native tissue repair, such as a multi-phase multi-cellular scaffold or plug[13,14], others have attempted to engineer graded interfaces with one cell type[15] or have engineered a whole multiphasic tissue from end to end with the goal of implantation to the injured site[16,17]. However, synthetic biomaterials could not fully simulate the original structure and matrix components of enthesis, and tendon/ligament constructs lacking bony interfaces are more prone to failure[18,19].

During the past decade, there has been increasing interest in creating biological scaffolds composed of extracellular matrix (ECM) derived from the decellularization of tissues or organs. The natural ECM is a complex network of proteins and polysaccharides forming an intricate meshwork within tissue that interacts with the resident cells to regulate cell behavior, such as migration, proliferation and differentiation[20]. Studies of skeletal muscle engineering have suggested that biologic scaffolds derived from site-specific homologous tissues may be better suited for constructive tissue remodeling than non-site specific tissue sources[21,22]. The use of decellularized ECM from donor tissue has been utilized in the repair of skin[23], bladder[24], heart valve[25] and small intestinal submucosa[26]. Decellularized enthesis ECM may provide a natural three-dimensional scaffold with tissue specific orientations of ECM molecules. These scaffolds are a first step towards enthesis tissue engineering for reconstruction of dysfunctional entheses. Decellularization protocols for bone, tendon, cartilage, and skeletal muscle have been widely discussed[27]. However, to our knowledge there is no efficient protocol for decellularization of Achilles tendon entheses with preservation of their biomechanical properties.

Chemical decellularization is a method that primarily uses chemicals to lyse and remove the cells and their components from the surrounding ECM. Chemicals frequently used for decellularization include sodium dodecyl sulfate (SDS), *t*-octylphenoxypolyethoxy-ethanol (Triton X-100), and tri-*n*-butyl phosphate (TnBP)[28]. Various formulations of DNases and RNases are also commonly used to remove nucleic acids from the material[29]. Single-reagent methods have been investigated for decellularization of articular cartilage, and 2% SDS or 3% Triton X-100 have been considered to be the most effective agents[30,31]. However, sample treatment time was seven days, thus the time efficiency was low[30,31]. A variety of detergent combinations were investigated to decellularize thick tendon samples, and it was suggested that two reagents might exhibit better decellularization with preservation of the natural tissue structure than single reagent[32]. Furthermore, decellularization was found to be significantly more effective when physical methods were included in the decellularization protocol, such as freeze-thaw cycles[33], ultrasound[34] or perfusion[35]. However, currently no standard treatment method is available for enthesis decellularization.

Here, we investigated the decellularization effect of combinations of chemical detergents with physical treatments, and established a novel standardized, time-efficient, and reproducible protocol to decellularize whole enthesis ECMs. Porcine Achilles tendon entheses were used due to high homology to human tissue. Decellularization efficiency was investigated by quantitative analysis of remaining cells, DNA analysis, and biomechanical characterization. Washing under 200 mmHg hydrostatic pressure with 0.5% SDS + 1% Triton X-100 for 48 h efficiently decellularized 2 mm thick entheses with preservation of matrix structure and biomechanical features. The presented protocol can be used to efficiently decellularize entheses as potential scaffolds for tissue engineering applications such as reseeding with mesenchymal stem cells to investigate differentiation and healing processes.

## Materials and methods

### Experimental design

This study used a two-phase approach. In study 1, different concentrations of combinations of two chemical detergents were examined at two treatment times (Table 1). Then, several physical methods were combined with the optimal chemical treatment from study 1, including freeze-thaw, ultrasound, perfusion, and hydrostatic washing (Table 2). A perfusion device and a hydrostatic washing system were developed for study 2. Cell counting, DNA assays, histological staining, and biomechanical tests were performed to evaluate the decellularization efficiency and structural preservation of treated groups.

**Table 1:** Chemical treatments for decellularization in study 1.

Group	SDS (w/v)	Triton-X (v/v)	Time
Control	-	-	
1	0.25%	0.5%	48 h
2	0.5%	1%	48 h
3	1%	1%	48 h
4	0.25%	0.5%	72 h
5	0.5%	1%	72 h
6	1%	1%	72 h

**Table 2:** Physical treatments investigated in study 2.

Group	Chemical treatment	Physical treatment	Time
7	0.5% SDS + 1% Triton X-100	Freeze-thaw: 3 x 30 min, Ultrasound: 3 x 10 min	48 h
8	0.5% SDS + 1% Triton X-100	Perfusion under 100 mmHg pressure	48 h
9	0.5% SDS + 1% Triton X-100	Perfusion under 200 mmHg pressure	48 h
10	0.5% SDS + 1% Triton X-100	Washing under 200 mmHg hydrostatic pressure	48 h

### Sample preparation

Porcine samples were obtained from a local abattoir in Munich, Germany. Fresh Achilles tendons with attached calcaneus were harvested from 6-month-old pigs (n = 10). All surrounding tissues were carefully removed using scalpels and frozen at -20°C. Frozen samples were cut into 2 mm thick slices using a band saw (300CL, EXAKT, Germany) with a diamond-coated stainless steel saw band of 0.4 mm in thickness, and then cut into 30 mm long and 5 mm wide samples using scalpels. The samples were washed in phosphate-buffered saline (PBS) to remove excess blood and frozen at -20°C in PBS containing 5% penicillin and streptomycin until processing, but for no longer than 1 week.

### Decellularization

#### Study 1

Different concentrations and treatment times used to optimize chemical treatment conditions are given in Table 1. Samples were treated in 50 ml sterilized plastic tubes with 40 ml chemical detergents on a shaker (Polymax 1040, Heidolph, Germany) at 20 rpm and room temperature with exchange of detergents every 24 h. For each concentration combination, incubation time of 48 h and 72 h were evaluated. Untreated samples were used as control (n = 4).

After chemical decellularization, samples were washed 3 × 30 min in distilled water at 4°C. Subsequently, samples and control group were incubated in PBS containing 100 μg/ml DNase (Sigma-Aldrich, USA) at 37°C for 24 h, covered with PBS, and stored at -20°C for evaluation of decellularization efficiency and biomechanical characterization.

#### Study 2

For study 2, physical methods (freezing and thawing, ultrasound, perfusion, hydrostatic washing) were combined with the optimal concentration of chemical reagents identified in study 1, and chemical treatment time was limited to 48 h (Table 2).

#### Freeze-thaw and ultrasound

Samples (n = 4) were subjected to three freeze-thaw cycles. After the first thaw in PBS at room temperature, samples were shock-frozen in liquid nitrogen in a hypotonic buffer (10 mM Tris, 2.7 mM EDTA, Sigma-Aldrich, USA) for 10 min and then thawed in PBS at room temperature for 10 min. The hypotonic buffer was replenished after the second thaw. Sequentially, ultrasound treatment was performed 3 × 10 min at 37 kHz at room temperature using Elmasonic S 60(H) (Elma Schmidbauer GmbH, Germany). After washing 3 × 30 min in PBS, the samples were incubated with 0.5% SDS + 1% Triton X-100 for 48 h as described in study 1.

#### Perfusion

Enthesis samples (n = 8) were clamped by a prefabricated mold and inserted into the inner cavity of a specially-designed perfusion tube. Then, the tube was installed to a custom-made perfusion device based on a previous set-up[36], and the perfusion was performed with 0.5% SDS + 1% Triton X-100 for 48 h under a pressure of 100 mmHg (n = 4) or 200 mmHg (n = 4) at room temperature.

#### Hydrostatic washing

Samples (n = 4) were incubated with 0.5% SDS + 1% Triton X-100 in syringes that were connected with a pump under a pressure of 200 mmHg. The syringes were placed on a shaker (Polymax 1040, Heidolph, Germany) at 20 rpm and room temperature with the exchange of detergents every 24 h.

Following the physical decellularization treatments, samples were washed, DNase digested and stored at -20°C as described in the protocol of study 1.

### Histological analyses

The decellularized samples (n = 4 for each group) and control samples (n = 4) were cut into 8 μm thick sections along the longitudinal direction using a cryostat microtome system (Thermo Scientific Microm, HM560, Germany) with -21°C objective temperature. Sections were stained using hematoxylin-eosin staining kit (CARL ROTH, Germany) and Masson-Goldner’s trichrome stain kit (CARL ROTH, Germany) following the manufacturer’s instructions to detect the cellular components and collagen fibrous structures, respectively.

### Cell quantification

Cell counts were performed on three randomized regions of interest (ROI = 400 × 300 μm^2^) per tendon, bone, and interface area in mosaiX pictures obtained using a Zeiss AxioObserver Z1 combined with Zeiss AxioVision software, thus a total number of nine ROIs per hematoxylin-eosin stained sample were counted. Cell density (n/mm^2^) was defined as cell count number divided by ROI area for different regions or total area for the whole sample, respectively. Decellularization efficiency was described by the percentage of cells removed from each treatment group compared with the control group. Mean ± standard deviation (SD) was calculated for all data.

### DNA assay

After decellularization, cryosections (n = 4 for each group) were collected on dry ice and weighed. DNA was extracted from tissue cryosections according to the manufacturer’s protocol of the DNeasy Blood & Tissue kit (QIAGEN, Netherlands). The DNA concentration was measured using a Nanodrop 2000 (MaestroNano, USA), and tissue DNA content was calculated according to the DNA concentration and sample weight (ng DNA/mg tissue). The DNA contents of treated groups were compared to the DNA contents of control groups.

### Biomechanical testing

Samples were treated according to group 5, group 10, and control group protocols (n = 7 each) and cut into samples of cross-sectional area 2 × 5 mm^2^ and a length of ≥ 30 mm using a scalpel. All specimens were then wrapped in PBS-soaked filter papers and allowed to thaw and equilibrate to room temperature for at least 2 h prior to mechanical testing.

**Fig 1:**
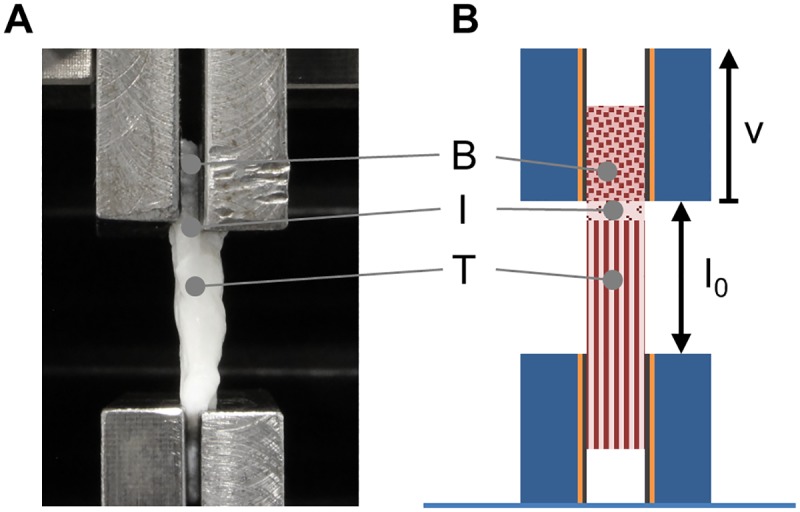
Biomechanical characterization of decellularized enthesis scaffolds. (A) Decellularized enthesis samples as well as control samples were biomechanically tensile tested in a customized set-up. (B) Tendon and bone part were clamped to the testing device and strain was applied at a constant speed ramp until failure of the specimen. B, I, T represent bone, interface region, and tendon, respectively.

Biomechanical testing was performed on an uniaxial test system (zwicki 1120, Zwick/Roell, Germany; load cell: KAF-Z, 2.0 mV/V, 2.5 kN, A.S.T., Germany). The samples were placed between two standard tensile test clamps: the bone part was attached to the upper clamp and the tendon part to the lower clamp with an initial tendon free length l_0_ of 20 mm (Fig 1). To avoid constraining shear and torsional loading, a passive bearing device was placed between the upper clamp and the traverse. The bearing device was able to freely move in the plane perpendicular to the loading axis to avoid shear forces as well as avoiding torsional moments in the loading axis. To apply strain to the specimen, a constant speed ramp of 0.5 mm/min was applied until failure of the specimen.

To evaluate three biomechanical parameters of the described specimen (maximum force, Young's modulus, and maximum elongation), force and displacement channels were recorded, as well as the individual geometrical values of the specimen to determine the initial cross section of each specimen.

### Statistical analysis

Statistical analysis was conducted using the software PRISM 6 (GraphPad, USA). The treated groups were compared to evaluate decellularization efficiency. The results were expressed as mean ± SD. Differences between groups were assessed using one-way analysis of variance (One-way ANOVA), followed by Tukey’s test for multiple comparisons. A p-value < 0.05 was considered statistically significant (* p<0.05, **^/##/+^ p<0.01, ***^/###/§^ p<0.001).

## Results

### Histological analysis and cell quantification

The amount of remaining cells after decellularization was evaluated for the three regions (tendon, interface, bone) of H&E stained entheses. Cell densities were calculated per region and per entire sample. The decellularization efficiency was determined by the ratio of residual cells to cells in the control.

**Fig 2:**
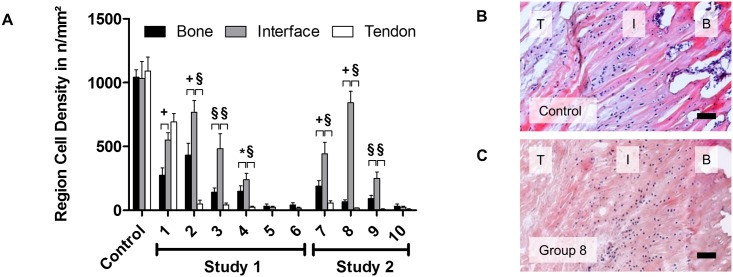
Regional difference of cell density in bone, interface, and tendon region after decellularization. (A) Cell density in bone, tendon, and interface region in decellularized samples from study 1 and 2 groups. For each group, cell density in the interface region was compared to cell density of the other two regions (* p<0.05, ^+^ p<0.01, ^§^ p<0.001). (B) H&E staining for control group; cells are distributed throughout the three regions. (C) Cell density reduction by decellularization differs between the regions; cells located in the interface region partially withstand the decellularization process. Scale bars correspond to 100 μm. T, I, B represent tendon, interface region, and bone, respectively.

All three regions (bone, interface zone, and tendon) showed high amounts of cells without treatment. The cell density was 1042±58 cells/mm^2^, 1033±133 cells/mm^2^ and 1092±108 cells/mm^2^ respectively without significant difference between the regions before the treatment (Fig 2A and 2B). However, the decellularization efficiency differed between the three regions. The amount of remaining cells after decellularization was significantly higher in the interface region than in the bone and tendon regions, in groups 2, 3, 4, 7, 8, and 9 (Fig 2A and 2C; * p<0.05, + p<0.01, § p<0.001). In group 5, 6, and 10, cells were completely removed from all three regions after the decellularization treatment.

**Fig 3:**
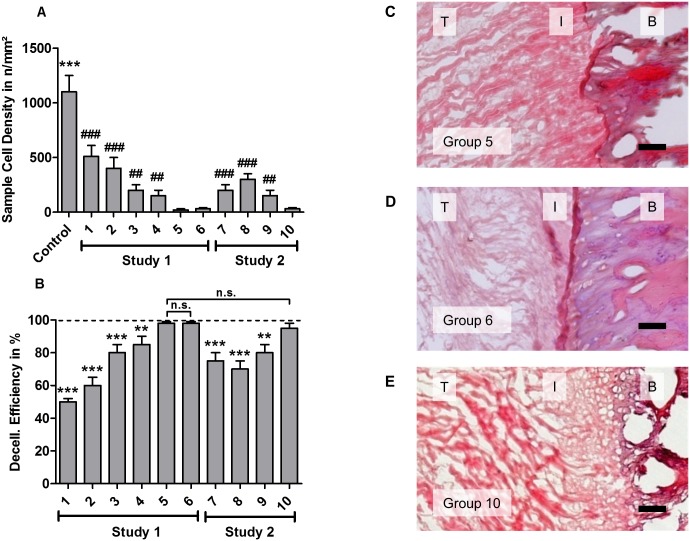
Decellularization efficiencies of study 1 and study 2 treatments. (A) Cell densities (mean±SD; averaged over sample) were calculated for each treated group and compared with control and group 5, which showed the lowest cell density after treatment (*compared with control *** *p*<0.001; ^#^compared with group 5, ^##^ *p*<0.01, ^###^ *p*<0.001,). (B) Decellularization efficiency was defined as the ratio of removed cells and compared with group 5 (** *p*<0.01, *** *p*<0.001). (C, D, E) Cells were efficiently removed from all three regions of the sample in group 5, 6, and 10. Scale bar = 100 μm. T, I, B refer to tendon, interface region, and bone, respectively.

In study 1, decellularization efficiency showed positive correlation with detergent concentration and treatment time (Fig 3A and 3B). Group 5 and group 6 treatments resulted in a high decellularization efficiency of 98% (Fig 3B, 3C and 3D). Since a lower SDS concentration was used in group 5 (0.5% SDS + 1% Triton-X 100 for 72 h), it was determined to be the most efficient chemical protocol for enthesis decellularization. Thus, this combination of SDS and Triton concentrations was used for study 2.

Study 2 showed that incubating with 0.5% SDS + 1% Triton X-100 under 200 mmHg hydrostatic pressure (in group 10) enabled complete removal of cells in the interface within 48 h (Fig 3E). The decellularization efficiency of group 10 was significantly higher than efficiencies of the other groups in study 2, but not significantly higher than group 5 efficiency.

**Fig 4:**
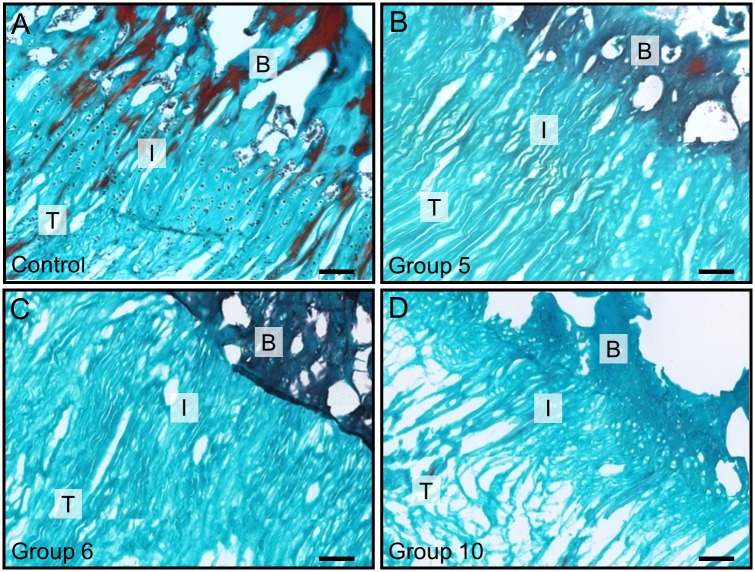
Masson’s trichrome staining for the collagen structure of entheses. (A) Control group showing collagen fibers with cells embedded in between. (B, C, D) Collagen alignment of treated samples for group 5, 6, and 10, respectively. Group 10 showed loosened collagen structure and partial ruptures. Cells were completely removed in B, C, and D. Scale bar = 100 μm. T, I, B refer to tendon, interface region, and bone, respectively.

Collagen structure integrity was assessed for groups that showed high decellularization efficiency using Masson’s Trichrome staining (Fig 4). In group 5, structural integrity of the collagen matrix was very high compared to the control group (Fig 4B). Group 6 showed partially loosened matrix structure with small gaps (Fig 4C). Structural integrity of group 10 samples was lowered, exhibiting disordered fibers and widened interfibrillar gaps (Fig 4D).

### DNA assay

**Fig 5:**
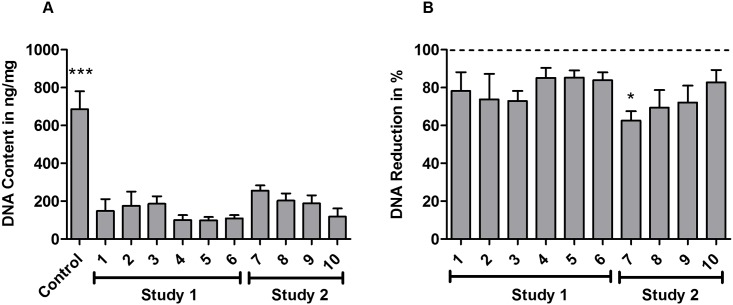
Decellularization treatments result in statistically significant reduction of DNA content. (A) DNA quantification for all groups, described in ng DNA per mg tissue. Statistically significant difference is indicated as *** p<0.001 compared with each treatment group. (B) DNA reduction efficiency (%) in decellularized groups. Values are expressed as mean±SD (n = 4 per group). All groups were compared with each other regarding statistically significant differences. Statistically significant difference of group 7 compared with the 72 h treatment groups (groups 4, 5, 6) was marked with * (p<0.05). The difference between all other groups was not statistically significant.

DNA content was investigated after decellularization treatment (Fig 5). All treatment groups exhibited a statistically significant reduction in DNA versus the control group (Fig 5A, p<0.001). Samples that underwent decellularization treatment for 72 h (study 1) showed lower DNA content than samples that were treated for 48 h (Fig 5A and 5B).

### Biomechanical testing

**Fig 6:**
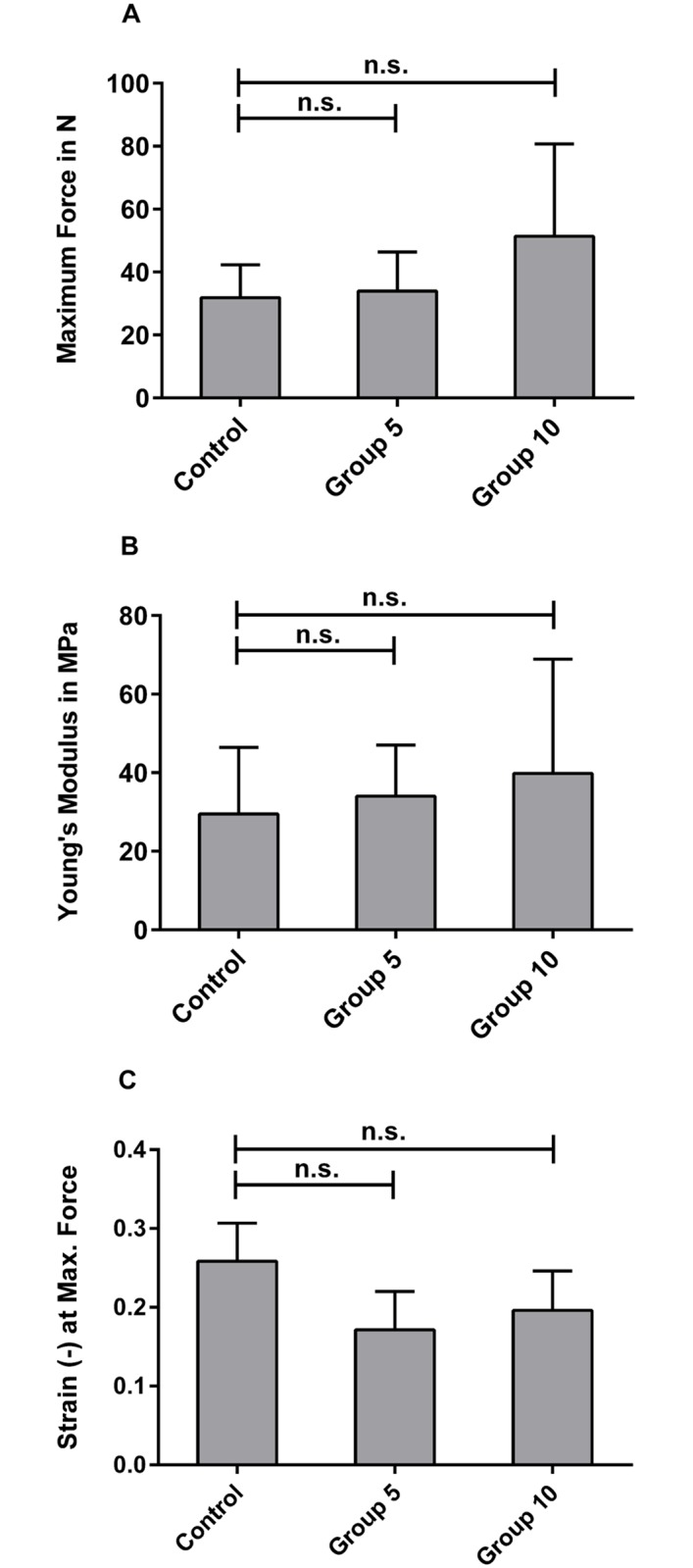
Tensile testing showed no statistically significant differences between samples decellularized using the two most efficient decellularization treatments (group 5 & 10) and untreated control. (A) Maximum loads, (B) Young's modulus, and (C) the elongation at the maximum force, noted as strain of control group (n = 6), group 5 (n = 7), and group 10 (n = 5); no statistically significant differences were observed between the groups.

Samples in control group (n = 7), group 5 (n = 7), and group 10 (n = 7) were subjected to tensile testing (Figs 1 and 6). Group 10 exhibited a higher maximum load (51.4±26.2 N) compared to the treated samples of group 5 (33.9±11.5 N) and the control group (31.9±9.5 N). The same trend was also observed for the Young’s modulus (group 10 39.8±26.0 N; group 5 34.0±12.0 N; control 26.5±15.5 N) and the maximum elongation in strain at the maximum force (group 10 0.2±0.04 mm; group 5 0.17±0.04 mm; control 0.26±0.04 mm). However, no statistically significant differences in maximum force, Young's modulus, or elongation values at maximum force were observed among the three groups (Fig 1A and 1B). Specimens that failed within the bone part were excluded from data evaluation (one sample in the control group and two samples in group 5).

## Discussion

The objective of this study was to establish an efficient protocol to decellularize porcine Achilles tendon entheses as scaffolds for enthesis tissue engineering. An ideal decellularized scaffold should have good biocompatibility for implantation and adequate mechanical strength for functional reconstruction. Since immunogenic antigens are distributed on the surface of cell membranes in the form of lipoproteins or glycoproteins, it is important that donor tissues are sufficiently decellularized to avoid immune rejection and inflammation[36].

Fibrocartilaginous enthesis have four zones of tissue: pure dense fibrous connective tissue, uncalcified fibrocartilage (UF), calcified fibrocartilage (CF), and bone. The UF and CF are avascular zones that are separated from each other by a basophilic line called the "tidemark"[37,38]; in this study "interface" represented both UF and CF zones. The interface region showed significantly more cells (^+/§^ p<0.01) than the tendon and bone regions after decellularization (Fig 2A and 2C). Therefore, the biggest challenge for whole enthesis decellularization was to remove cells from the fibrocartilaginous zone.

Previously, Triton X-100, as a nonionic surfactant, could permeabilize cellular membranes, solubilize membrane proteins, and extract DNA. Decellularization with Triton X-100 completely removed nuclear material in nerves, pericardium, and bones [39–41]. SDS, as an anionic surfactant, could lyse cells and denature proteins by disrupting noncovalent bonds. Decellularization with SDS removed cells in the meniscus, cornea and cartilage bone[29,42,43]. The decellularization effect was related to the organization of the material, as well as the concentrations of detergents. For example, Chan et al.[44] observed that many dead cells were left in the intervertebral disk with 0.1% SDS, whereas Xu et al.[31] reported that 0.5% SDS produced no cells in decellularized porcine annulus fibrosus.

On the contrary, exposing tissues to these agents for too long can alter the mechanical properties of the ECM. It is often advantageous to use several chemicals in a series of short wash cycles to increase the efficiency and reduce the time for which a tissue is exposed to any individual chemical[45]. This study was the first to evaluate the combination of SDS and Triton X-100 for enthesis decellularization. Study 1 showed that low dose chemical treatment (0.25% SDS + 0.5% Triton X-100 for 48 h in group 1) removed half of the cells (Fig 3). Decellularization efficiency increased with detergent concentration and treatment time.

Group 5 and group 6 showed highest decellularization efficiency (Fig 3B) with efficiencies of 98.1% ± 0.4% and 97.9% ± 0.7%, respectively. Statistically significant difference was observed between group 5 and group 6 compared to the other groups. No statistically significant difference was found between the group 5 and group 6. Since group 5 involved chemical treatment with lower concentrations than group 6, group 5 treatment (0.5% SDS + 1% Triton X-100) was considered to be the most efficient combination for chemical methods.

Physical treatment may contribute to decellularization efficiency[30,35,46–48], since they may rupture cell membranes and facilitate the transport of decellularization solution to the cells and cellular material from the tissue[27,49,50]. Here, we systematically evaluated the contribution of different physical approaches: freeze-thaw cycles, ultrasound, perfusion, and washing under pressure to decellularization efficiency. Chemical treatment with 0.5% SDS + 1% Triton X-100 for a limited exposure time of 48 h was set as baseline. Decellularization efficiency of group 7 (freeze-thaw/ultrasound) was 77.7% ± 6.3%, which was significant lower than chemical treatment only in group 5 (98.1% ± 0.4%, p<0.01, Fig 3B). Group 8 and group 9, perfusion at 100 mmHg and 200 mmHg, respectively, showed that decellularization efficiency increased with increased pressure (70.4% ± 3.1% to 88.8% ± 2.6%, respectively). However, perfusion decellularization efficiency was not sufficient to completely remove cells at the interface region. This may be caused by high water resistance and the avascular characteristics of fibrocartilaginous tissues.

Washing under 200 mmHg pressure was used to increase penetration of the dense tissue with decellularization agents. Group 10 showed that it was possible to shorten the treatment time from 72 h to 48 h while obtaining high decellularization efficiencies. Here, decellularization efficiency was 95.7% ± 2.2% with no statistically difference to that of group 5.

The native matrix microstructure of fibrous and fibrocartilaginous tissues is rather dense resulting in diffusion limitations of decellularization chemicals into deeper tissue zones. Thus, most tissue derived scaffolds are from processed tissues such as cartilage sheet sandwiches or cartilage particles[30,51–53]. In our study, decellularization was optimized to successfully decellularize intact, even 2 mm thick enthesis samples as macroscale tissue engineering scaffolds.

DNA is an important component to evaluate the decellularization effect[30,48,49]. In this study, DNase was used after every decellularization process, and quantitative analysis of DNA content was performed to identify differences between the treatments. The results demonstrated that all protocols had the capability to reduce the DNA content obviously compared with the control group (p<0.001, Fig 5A). Unlike the cell count results, the DNA contents of the treatment groups had no significant difference between each other at the same treatment time, no matter whether combined with physical methods (Fig 5B). However, the DNA content in 72 h treated groups was significantly reduced compared to 48 h treated groups (p<0.05, Fig 5B). It seemed that the amount of remaining DNA mainly depended on the detergent exposure time.

Another aspect of the study was to evaluate whether decellularization had destructive effects on the matrix. According to Masson staining, chemical-only treatment showed largely intact collagen structures with the exception of few local ruptures and slightly loosened collagen fibers in group 5 and group 6 (Fig 4B and 4C). In combination with physical treatment, localized ruptures and collagen gaps were observed (Fig 4D). Structure loosening may promote detergent penetration into fibrocartilage tissue, but biomechanical properties may also be influenced. Thus, we biomechanically characterized scaffolds from the two most promising groups (group 5 and group 10) as well as an untreated control group with regard to maximum force, Young’s modulus, and maximum elongation. No statistically significant difference of biomechanical properties of treated samples compared with the control group was observed (Fig 6). It may be due to a relatively low concentration (0.5% SDS and 1% Triton X-100) and short treatment time (48 h vs. 72 h in total) that biomechanical characteristics were retained. Cartmell et al. (2004) also report that decellularized patellar tendon grafts show similar biomechanical characteristics despite morphological changes in the tissue[54]. However, Woods et al. observed changes in tensile stiffness after decellularization of ACL-bone grafts[55]. Using several chemicals in a series of short washing cycles and reduced exposure times may thus increase efficiency and minimize destructive effects on scaffold structure and biomechanical properties.

The present study had certain limitations. First, collagen and glycosaminoglycan (GAG) contents were not measured in this study. Collagen and GAG content are the main components of the enthesis and play an important role in guiding cellular attachment, survival, migration, proliferation, and differentiation[56]. Xu et al.[57] reported no collagen loss in the decellularization process using Triton X-100, SDS, or trypsin. However, GAG content was reduced with decellularization and Triton X-100 was superior to the other treatments in retaining GAG content. Although the ideal decellularized enthesis scaffold should have collagen and GAG content close to that of the natural tissue, should have chondroinductive effects[58,59], and/or should be beneficial for cell signaling, some other previous studies indicated that a partial reduction in GAG content might be beneficial to create a less dense matrix that allows cell infiltration and migration[60]. Second, the investigation was performed in vitro. Hence, the biocompatibility of the natural scaffold in tendon-to-bone healing in vivo remains unknown. Most importantly, the recellularization potential of the decellularized enthesis scaffold and its application in animal implantation research should be investigated systematically in the future.

## Conclusion

This study established a practical and time-efficient protocol to decellularize porcine Achilles tendon entheses using a combination of two chemical detergents (0.5% SDS and 1% Triton X-100) with a physical method (washing under 200 mmHg hydrostatic pressure). The decellularization protocol was applicable also to very thick samples of 2 mm thickness. We showed that the interface region featured different decellularization properties than the adjacent tendon and bone regions; decellularization efficiency at the interface region was statistically significant reduced compared to tendon and bone. Here, we systematically established a protocol that did not only decellularize tendon and bone, but also the interface region of the Achilles tendon enthesis. Biomechanical testing showed no statistically significant difference between decellularized and control samples. The proposed decellularization strategy is applicable to decellularize tendon-bone interfaces as scaffolds for tissue engineering.
